# Evaluation of Hemoglobin Concentration of Cord, Capillary and Venous sampling in Neonates

**Published:** 2012-09-22

**Authors:** Z Eslami, R Ghilian, F Abbasi

**Affiliations:** 1 Department of Pediatrics, Hematology, Oncology and Genetics Research Center, Shahid Sadoughi University of Medical Sciences and Health Services, Yazd, Iran.; 2 Department of Internal Medicine, Shahid Sadoughi University of Medical Sciences and Health Services, Yazd, Iran.; 3 Department of Pediatrics. SSODAYClinic, Rafsanjan, Iran.

**Keywords:** Anemia, Hemoglobins, Capillaries

## Abstract

**Background:**

Anemia is one of the most common abnormalities in pediatric medicine. Considerable differences are seen in the peripheral blood indices of infants in developing countries. The aim of this study was to determine and compared blood hemoglobin concentration in three sites of sampling in neonate.

**Materials and Methods:**

1600 term and preterm new infant in three sites of sampling (cord, capillary and venous) were taken under investigation. All neonates were examined during first day. The methodology excluded patients with a high likelihood of receiving blood transfusions and those who had a diagnosis of neonatal anemia. Finally, obtained data were analyzed and then compared to other hematology results.

**Results:**

Mean hemoglobin value obtained from cord was 15.39+/- 5.39 SD and from capillary were 19.62+/- 5.75 SD and from venous were 17+/- 7.79 SD. Mean hemoglobin value of cord in term neonates were 15.4+/- 5.07 SD and pre term neonates were 14.77+/- 1.69 SD. (P=0.036) Mean hemoglobin value of capillary in term neonates were 19.63+/- 5.76 SD and pre term neonates were 18.85+/- 1.79 SD. (P=0.015) Mean hemoglobin value of venous in term neonates were 17.01+/- 7.81 SD and pre term neonates were 16.15+/- 1.76 SD. (P=0.012) There was a correlation between cord and capillary mean hemoglobin. (P=0.0235)

**Conclusions:**

The capillary samples had a higher mean hemoglobin concentration than other groups. There was no difference between these data with respect to the hemoglobin values for any groups.

## Introduction

Anemia is defined as a reduction in the number of circulating erythrocytes. Anemia is typically reported initially by automated red blood cell (RBC) indices. Clinicians usually check their suspicion of this disorder by referring to the normative hematological reference values for neonate in reference textbooks. The blood hemoglobin concentration is the most commonly performed of all clinical laboratory tests. Many articles describe reference ranges for hematocrit and blood hemoglobin concentration during the neonatal period. The standard values are used for all. Literature revealed that there is a limited difference of peripheral blood cell counts and indices of infants in developing countries (1, 2). 

The aim of this study was to determined hemoglobin concentration of new born infants and compared with standard reference range. In addition three site of sampling (capillary, venous and cord blood) were further compared to each other. 

## Materials and Methods

We determined mean hemoglobin concentration of 1600 neonate with standardized hematology analyzer from three hospitals in Yazd, Iran. Thirty one of parents did not endorse their testimonial. They were subcategorized by gestational age and sex and weight to account for the effects of both maturation and sex on hemoglobin levels. New infants were obtained for clinical use from neonate who had not received an erythrocyte transfusion and did not have associated conditions. Specifically, data were not included when the patient had a diagnosis of neonatal anemia, intra uterus growth retardation (IUGR) and small gestational age (SGA) or when the mother had the diagnosis of placenta previa or abruptio placenta, chronic hypertension and diabetes. We obtained samples from three sites (capillary, venous and cord blood). Capillary and venous samples obtained during the first day of life. Capillary samples obtained by skin prick, from the heel or toe. Because our data were collected from a single demographic area of Iran, they could not be representative of all Iranians. 

**Statistical analysis**

The mean Hemoglobin reference ranges for each group were extracted from the ADVIA120 hematologic analyzer data files (3) .The mean hemoglobin values for neonates delivered at term with weight greater than 2500 g from the ADVIA 120 subsample was compared to our study. Mean values were statistically analyzed using a Student’s t test. Statistical significance was set as P< 0.05. 

## Results

This was a cross sectional study. Of the 1600 new infants, 846 were males and 754 females.1558 delivered at term and 42 delivered at pre term (infants born between 38 to 42 weeks of gestation). In this study capillary samples had a higher mean hemoglobin concentration than simultaneously collected venous and cord samples. (Table I) 

Mean hemoglobin value that obtained from cord in male was 15.55+/- 6.74 SD and in female was 15.19+/- 1.58 SD (P=0.151). Mean hemoglobin value that obtained from capillary in male was 19.55+/-1.96 SD and in female was 19.66+/-8.04 SD (P=0.733). Mean hemoglobin value that obtained from venous in male was 17.18+/-10.5 SD and in female was 16.76+/-1.62 SD (P=0.348). When the data was compared between term and preterm group, the finding showed a significant difference in the mean hemoglobin between both groups. However mean hemoglobin concentration in term neonate was higher than preterm (P<0.05) (Table II). We compared the data for weigh of neonates, mean hemoglobin value that obtained from cord in neonate with lower than 2.5 Kg was 15.03+/-1.59 SD and in neonate between 2.5to 3.5 Kg was 15.38+/-4.67SD and in neonate with higher than 3.5 Kg was 15.47+/-6.32SD (P=0.845). Mean hemoglobin value that obtained from capillary in neonate with lower than 2.5 Kg was 19.65+/-1.82SD and in neonate between 2.5to 3.5 Kg was 19.64+/-6.97SD and in neonate with higher than 3.5 Kg was 19.5+/-1.83SD (P=0.978). Mean hemoglobin value that obtained from venous in neonate with lower than 2.5 Kg was 16.79+/-1.7 SD and in neonate between 2.5to 3.5 Kg was 16.81+/-1.64SD and in neonate with higher than 3.5 Kg was 17.46+/-1.45 SD (P=0.5) 

There was a correlation between cord mean hemoglobin and capillary mean hemoglobin. According to Pierson correlation factor, it was found significant communication between them. (P=0.0235) When cord hemoglobin increased, capillary hemoglobin increased. In this study 485 neonates were first child and 579 neonates were second and third child and 259 neonates were fourth child. We found that the mean hemoglobin value was higher in first child than others (15.6+/-6.01SD vs.15.38+/-6.04SD, 15.45+/-1.74SD), (P=0.915). Our finding confirmed that 0.1% of neonate’s hemoglobin concentration obtained from venous were anemia. (Lower than 13g/dl) and0.3% of neonate’s hemoglobin concentration obtained from cord and capillary were anemia (Lower than 14.5g/dl).

**Table 1:** The mean hemoglobin concentration according to gestational age

site of sampling	Number	Mean	S.D	Normal Limit
Cord	1569	15/39	5/06	15/39±0/128
Capillary	1569	19/62	5/75	19/62±0/145
Venous	1569	17	7/79	17±0/197

**Table II:** The mean hemoglobin concentration three sites of sampling according to gestational age

estational age	Term			Pre -term			p-value
site of sampling							
Cord	Number	Mean	S.D	Number	Mean	S.D	0/036
	1558	15/4	5/07	42	14/77	1/69	
Capillary	1558	19/63	5/76	42	18/85	1/79	0/015
Venous	1558	17/01	7/81	42	16/15	1/76	0/012

## Discussion

Anemia is one of the most common abnormalities encountered by general pediatricians. With the costs and restrictions of present-day health care, not only unnecessary anemia evaluation and treatment should be avoided but also prevent the misdiagnosis of anemia in Iranian new infant. Thus we decided to compare mean blood hemoglobin concentration in Iranian neonates with standard reference range.

 The current study represents that there is no significant differences between the age and sex group with respect to the normative hemoglobin values. The mean hemoglobin value obtained from cord was 15.4g/dl. The observed report corresponds with the report of ADVIA, in which the mean hemoglobin value was 15.9+/-1.86 SD (3). In a similar reference normal mean hemoglobin value obtained from cord was 14-22g/dl (4, 5). The mean hemoglobin value obtained from venous in our study was similar to other references (14.5-23 g/dl) (4).

The mean hemoglobin value obtained from capillary in the present study was 19.62g/dl which resemble the same as other reference (14.5-25g/dl) (4). In a study conducted by Edwin et al. in New York, showed that African American children and adolescents have lower mean hemoglobin values in compare to Whites generation (6). Other study in Nigerian town by Nduka et al. compared hematologic indices of 512 Africans and 196 Caucasians found marked differences between the two groups, with Caucasians having higher hemoglobin levels (8). Tc hernia employed 199 newborns in Bamako (Mali) succeeded to revealed that this group had lower hemoglobin levels than a control group of French newborns (1). 

In one study performed by Joplin et al. in Utah, could develop reference ranges for hematocrit and hemoglobin during the neonatal period (28 days) using very large sample sizes and modern hematology analyzers. They concluded that reference ranges was similar to those expected at sea level (8). The effect of gender on the initial hematocrit/hemoglobin was also previously unclear (9, 10). We found difference on the basis of gender but study of Joplin et al. did not show effect of gender regardless the hematocrit or hemoglobin. They confidently concluded correlation between the sexes (6). In preterm infants who are already born with a lower hemoglobin, is referred to as anemia of prematurity (AOP). The primary cause of AOP is the impaired ability to increase serum erythropoietin (EPO) appropriately in the setting of anemia and decreased tissue availability of oxygen (11, 12).

Similar to Joplin et al. we had a clear picture of physiology among the relatively healthy late preterm and term neonates who had not transfusions. In our study hemoglobin values of capillary beds in neonates were higher than those simultaneously of vascular and cord sources. This attributes to the study of Oh and Linda who suggested that at least in part, to the larger size of erythrocytes in neonates, which may flow more slowly through capillary beds and lead to hem concentration of capillary blood (11).

While heel lancing is technically simple, however produces tissue trauma, and is irreproducible, Therefore squeezing the heel repeatedly is often required getting sufficient blood for sampling. Heel lance blood samples, therefore, can be influenced by local injury and hemolysis. In a retrospective comparative study, Moe et al, demonstrated that from 41 infants, 25 were found to be anemic based on determinations performed on cord blood sample, whereas only 14 were considered anemic on the basis of the results of capillary sample analysis (12).

## Conclusions

Hematocrit and blood hemoglobin concentration are measured frequently for diagnosis of anemia. We found no difference in mean hemoglobin value for each site of sampling as compared to reference normative mean hemoglobin value and there seems to be statistically significant difference between mean hemoglobin concentration in term and preterm group in three sites sampling .These data suggest that use of the reference hemoglobin values will help preventing the misdiagnosis of anemia in Iranian neonate and thereby minimize unnecessary hematological workups and treatment.

## References

[1] Dialio DH, Sidibe S (1994). Prevalence de l’anemie du nouveaune au Mali. Cah Sante.

[2] Harthoorn-Lasthuizen EJ, Lindemans J, Langenhuijsen MM (2001). Does iron-deficient erythropoiesis in pregnancy influence fetal iron supply?. Acta Obstet Gynecol Scand..

[3] Carlo Brugnara, Orah S Platt, Orkin SH, Nathan DG, Ginsburg D, Thomas Look A, Fisher DE, Lux SE (2009). The Neonatal Erythrocytes and Its Disorders. Hematology of Infancy and Childhood.

[4] Robins EB, Blum S (2007). Hematologic reference values for African American children and adolescents. Am J Hematol.

[5] Nduka N, Aneke C, Maxwell-Owhochuku S (1988). Comparison of some haematological indices of Africans and Caucasians resident in the same Nigerian environment. Haematologia (Budap).

[6] Jopling J, Henry E, Wiedmeier SE, Christensen RD (2009). Reference ranges for hematocrit and blood hemoglobin concentration during the neonatal period: data from a multihospital health care system. Pediatrics.

[7] Burman D, Morris AF (1974). Cord haemoglobin in low birth weight infants. Arch Dis Child.

[8] Christensen RD (2000). Expected hematologic values for term and preterm neonates. Hematologic Problems of the Neonate.

[9] Stockman JA 3rd, Graeber JE, Clark DA, McClellan K, Garcia JF, Kavey RE (1984). Anemia of prematurity: determinants of the erythropoietin response. J Pediatr.

[10] Brown MS, Phibbs RH, Garcia JF, Dallman PR (1983). Postnatal changes in erythropoietin levels in untransfused premature infants. J Pediatr.

[11] Oh W, Lind J (1966). Venous and capillary hematocrit in newborn infants and placental transfusion. Acta Paediatr Scand.

[12] Moe PJ (1967). Umbilical cord blood and capillary blood in the evaluation of anemia in erythroblastosis foetalis. Acta Paediatr Scand.

